# Dealing with Induced Fit, Conformational Selection, and Secondary Poses in Molecular Dynamics Simulations for Reliable Free Energy Predictions

**DOI:** 10.1021/acs.jctc.3c00867

**Published:** 2023-12-01

**Authors:** Piero Procacci

**Affiliations:** Dipartimento di Chimica “Ugo Schiff”, Università degli Studi di Firenze, Via della Lastruccia 3, 50019 Sesto Fiorentino, Italy

## Abstract

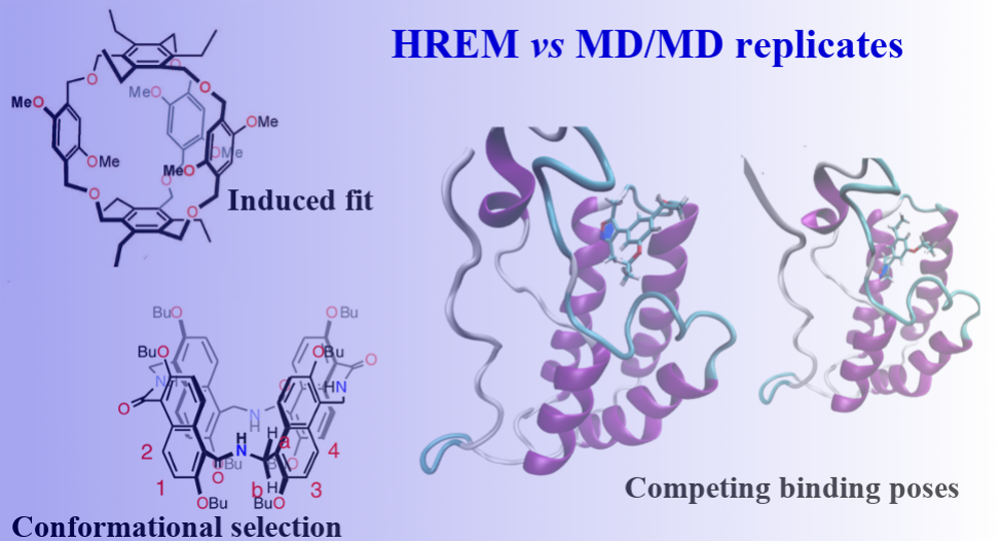

In this study, we have tested the performance of standard molecular dynamics (MD) simulations, replicates of shorter standard MD simulations, and Hamiltonian Replica Exchange (HREM) simulations for the sampling of two macrocyclic hosts for guest delivery, characterized by induced fit (phenyl-based host) and conformation selection (naphthyl-based host) and of the ODR-BRD4(I) drug-receptor system where the ligand can assume two main poses. For the optimization of the HREM simulation, we have proposed and tested an on-the-fly iterative scheme for equalizing the acceptance ratio along the replica progression at a constant replica number resulting in a moderate impact of the sampling efficiency. Concerning standard MD, we have found that, while splitting the total allocated simulation time in short MD replicates can reproduce the sampling efficiency of HREM in the phenyl-based host and in the ODR-BRD4(I) complex, in the naphthyl-based macrocycle, characterized by long-lived metastable states, enhanced sampling techniques are the only viable alternative for a reliable canonical sampling of the rugged conformational landscape.

## 1 Introduction

Identification of prospective drugs via a computational approach represents a major challenge in drug discovery. More specifically, elimination of false positive produced in high throughput virtual screening campaigns is still one of the key problems in the early stages of drug design.^1^ In a hierarchical, funnel-shaped computational pipeline, hits from cost-effective docking or supervised machine learning approaches, before undergoing wet-lab assessment, are often purged of false positives using expensive molecular dynamics (MD) techniques with a full atomistic description of the system.^2−6^

Most of the current MD applications for computing the ligand–receptor binding affinities are based on the so-called alchemical approach using Free Energy Perturbation (FEP),^7,8^ whereby the buildup of ligand-environment interactions is assessed in the two legs of a thermodynamic cycle (the ligand in the bulk and in the bound state) by constructing a series of intermediate connecting alchemical states. The binding free energy between the physical end states in the two legs of the cycle is recovered as a sum of free energy difference between nonphysical alchemical states using free energy perturbation^9^ and the Bennett acceptance ratio^10^ or thermodynamic integration.^11^ The *absolute* binding free energy can hence essentially be computed as a difference between two solvation free energies, namely that of the ligand in bulk solvent and in the bound state.^8,12^ Alchemical techniques can be likewise adapted to evaluate the relative binding free energy between two *congeneric* compounds by gradually transmuting one compound into the other in a series of “chimeric” states in the two legs of the cycle.^3,13^

Despite progress in the past decade of algorithms, force fields, and methods^14−16^ and despite the spectacular growth of computer power recently boosted by the advent of GPU-CPU heterogeneous high performing computing (HPC) architectures, alchemical techniques based on FEP still face daunting computational challenges, mostly related to the difficulties in achieving a stationary (converged) sampling^3,17,18^ in conformationally complex drug receptor systems. In this respect, molecular recognition is widely thought to be driven by two main mechanisms, namely the induced-fit^19^ model or the conformational selection paradigm.^20^ According to the former, the protein adapts its conformation upon ligand binding, switching from an *apo* (unbound) to a *holo* (bound) form, while in the latter case, believed to be the cornerstone of the allosteric effect,^21^ binding is possible only when the protein is in one particular conformation among several metastable states in chemical equilibrium.

Structural differences between the *holo* and the *apo* forms in the induced fit can be difficult to address with FEP-based alchemical approaches due to the long time scale typical of these conformational transitions. Binding through conformational selection relies on the identification of the binding metastable state of the protein, sluggishly exchanging between several alternative long-lived conformations, a task that can be achieved only resorting to enhanced sampling techniques such as Hamiltonian Replica Exchange^22−24^ (HREM) or Metadynamics.^25,26^

Recently, in the context of supramolecular chemistry, two paradigmatic macrocyclic receptors have been designed for induced fit^27^ and conformational selection^28^ ligand selectivity. The first compound is a structurally flexible macrocyclic cage characterized by two distinct rapidly exchanging conformations due to the flipping of the three phenyl sidewalls connected by methylene bridges. The second compound has a cage with four naphthalene sidewalls whose flipping defines several different, slowly interconverting conformations that can be distinguished in NMR spectra. In this paper, at fixed CPU/simulation time allocation, we compare the efficiency of HREM and standard MD simulations in these conformationally challenging host molecules. In particular, the sampling effectiveness of standard MD simulations, at constant CPU investment time, is tested using (i) a single long trajectory and (ii) replicates of independent short trajectories. For the HREM simulations, we compare, at constant replica number and constant CPU time, the performance of an on-the-fly iterative scheme for acceptance ratio equalization with a commonly adopted stationary scaling protocol.

Convergence of vanilla MD, MD replicates, and HREM with and without acceptance ratio equalization has been further assessed and compared in a biologically relevant system, consisting in the complex of a 3-(trifluoromethyl)phenyl]-5-isoxazolamine compound (ORD) with the first bromodomain-containing protein 4 (BRD4). Such a peculiar system is characterized by two distinct ligand poses, whose probability ratio is strongly affected by the alchemical state of the ligand.

The crucial importance, revealed in our study, of enhanced sampling when dealing with common molecular recognition mechanisms, such as induced fit and conformational selection, or common phenomena, such as pose competition in a drug-receptor system, has important implications in the setting up of an automated, efficient, and reliable alchemical protocol for a high-level screening of docking hits for false positive elimination in drug discovery projects.

## 2 Theoretical Background

Typically, events such as conformational transitions in proteins or flexible ligands with large torsional barriers around rotatable bonds occur abruptly, involving large changes of the relevant collective coordinate (e.g., a dihedral angle) in a *subpicoseconds* time scale. These rapid transitions, triggered by random collision or concerted fluctuations, are nonetheless *rare*, in the sense that few of them can be observed in a *single* molecule or flexible ligand on a time scale from nanoseconds to milliseconds.^29^ In many-molecules systems, rare events become frequent when we test their generic reduced probability of occurrence on *any one* of the molecules.^30^ So, while observing a rare event on *some* molecule is common in a thermodynamic system, it is a matter of pure luck if one is monitoring the behavior of one single molecule during a typical MD simulation lasting a few tens of ns. Recently, the advent of GPU and the porting of MD algorithms to GPU architectures have boosted the speed of popular MD codes by more than 1 order of magnitude.^31−33^ A typical solvated drug-receptor system in periodic boundary conditions (50 K-100 K atoms) can now be run for hundreds of nanoseconds per day on a high-end heterogeneous HPC platform. However, in many cases, this time span can be insufficient for obtaining statistically converged simulations since we are dealing with a single-molecule system and not with a thermodynamic ensemble. Unconverged runs on single complexes typically plague the final outcome of the prediction using FEP or TI-based alchemical approaches for binding free energy calculations.^34,35^ The sampling problem is patently exposed when results are found to critically depend on similar starting conditions so that in general *replicates* of costly alchemical free energy calculations must be often carried to deliver a reliable prediction with a credible confidence interval.^36−40^

Enhanced sampling techniques are often used to overcome these sampling-related issues. HREM is certainly one of the most effective advanced MD methods for simulating systems with rugged conformational landscapes.^22−24,41,42^ HREM is generally engineered to be *collective coordinate agnostic*, scaling the full potential energy of the system or a part of it as in the so-called solute tempering scheme.^23,24,42^ This is a significant advantage in drug-receptor systems where we do not generally know *a priori* which collective variables are involved in the induced fit or regulation of allosteric effects.

In an HREM simulation, several MD simulations are run in parallel on *n* states or nodes with gradually scaled potential energy, up to a minimum scaling factor *s*_*n*_ = *s*_min_ such that
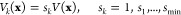
1Exchanges between scaling factors are periodically attempted, with an acceptance probability based on a Metropolis criterion satisfying the detailed balance condition. In so doing, we observe a random diffusion of the scaling factors (or inverse “temperatures” *s*_*k*_β), while the HREM simulation proceeds. A *replica* is hence an MD trajectory spanning various Hamitonians with different scaling factors, providing a mechanism for transmitting conformations sampled in states with strongly scaled potential energy to the *target* (unscaled) state with *s* = 1 and the correct Boltzmann weight. The acceptance probability *P*_acc_(*k*, *k*′) for the *k* ↔ *k*′ exchange depends on the overlap of the corresponding potential energy distributions,^23^ so that exchanges are normally attempted between *contiguous* replicas. In this case, we use the shorthand notation *P*_acc_(*k*) to denote the acceptance probability for the exchange *k* ↔ *k* + 1.

The needed number of nodes *n* in an HREM simulation depends on the total number of degrees of freedom *N* involved in the scaling. As the mean potential energy *E̅* and its variance  are extensive functions, the larger is the number of degrees of freedom *N* involved in the scaling, the more states will be necessary for a significant overlap of contiguous distributions with .^30^

The tuning of an HREM simulation for optimal sampling efficiency is by no means a trivial task. As shown in ref.,^43^ the exchange attempt frequency (EAF), one of the parameters of an HREM simulation, should be chosen as high as possible (typically 0.03–0.05 ps^–1^) provided that the communication cost of the attempted exchange in a parallel execution is small as it happens when we exchange scaling factors rather than configurations. High *P*_acc_(*k*) are in principle desirable, but they are costly as they involve a large *n*. Like in standard Monte Carlo, it is generally believed that an optimal (and balanced) *P*_acc_(*k*) in systems with kinetics traps should be in the range 0.2–0.5. Balancing the acceptance probabilities *P*_acc_(*k*) *may*, in general, result in a smoother replica diffusion and in a shortening of the round trip times (RTTs). On the other hand, as noted in ref.,^44^ well balanced acceptance ratios along the states progression do not guarantee an optimal *flux* in HREM simulation. The upward flux can be measured as
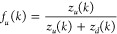
2where *z*_*u*_(*k*) and *z*_*d*_(*k*) are the number of visits to state *k* by replicas coming from state 1 (the target state) and from state *n*, respectively. Due to replica conservation, we have *f*_*d*_(*k*) = 1 – *f*_*u*_(*k*) where *f*_*d*_(*k*) is the downward flux. Ideally, the optimal flux is obtained when *f*_*u*_(*k*) = 1 – *k*/*n* and *f*_*d*_(*k*) = *k*/*n*, leading to a *linear* flow distribution with constant transition probabilities. In principle, in an HREM simulation, we should try to adapt the scaling ladder to reach such a linear regime for the flux. Flow optimization in HREM is prohibitively costly as it requires long adaptive enhanced sampling simulations to estimate to flux, since many visits to state *k* are necessary to get a statistically reliable *f*_*u*_(*k*). An alternative less demanding estimate is evaluated, rather than directly using slowly convergent eq 2, using the approach described in ref.^44^ based on the mean first passage times (MFPTs) *within* the scaling factor ladder (see eqs 6–11 of ref.^44^). In the latest ORAC distribution,^45^ we provide a simple bash script, replica_flux.bash, to compute the flux in HREM using the MFPT approach proposed by Nadler et al.^44^

While flow optimization, whether using eq 2 or the estimate based on the MFPT, is costly, balancing of the acceptance ratio *P*_acc_ can be achieved rather quickly with adaptive HREM scaling. Balanced *P*_acc_ avoids any possible “bottleneck” in the HREM simulation. Bottlenecks may significantly slow down convergence and give rise, in the worst case scenario, to regions of noncommunicating replicas due, e.g., to some sharp transition as a function of the scaled potential, typical of rare events (e.g., transition between torsional-driven conformational states).

We may hence try to optimize our HREM by balancing the *P*_acc_(*k*) value on the fly. To this end, starting from the traditional scaling protocol based on the geometric progression,
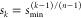
3whereby overlap between contiguous replica is maximized assuming that the system may be described by an ensemble of harmonic oscillators,^23,46,47^ we propose to periodically reset, with a frequency ω ≪ *EAF*, the scaling ladder according to the iterative scheme
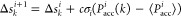
4where *Δs*_*k*_^*i*^ = *s*_*k*_^*i*^ – *s*_*k*+1_^*i*^ is the *n* – 1 *positive* differences between contiguous scaling factors for the *n* states at iteration *i*, *P*_acc_^*i*^(*k*) is the acceptance probability for the *k* ↔ *k* + 1 exchange at iteration *i*,  is the mean acceptance probability at iteration *i*, and σ_*i*_ is the corresponding standard deviation. For an optimal balance of *P*_acc_(*k*), the final value of σ_*i*_ should be as small as possible. According to eq 4, the differences *Δs*_*k*_^*i*+1^ are corrected by a factor that is strictly proportional to the product of standard deviation σ_*i*_ and the difference between the actual value of *P*_acc_(*k*)^*i*^ and the mean acceptance ratio. As the sum of the differences, ∑_*k*_^*n*–1^*Δs*_*k*_^*i*^ = 1 – *s*_min_ is invariant due to the fact that at each iteration
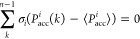
5The update scheme of eq 4 is designed to constantly reduce the variance σ_*i*_^2^ thereby gradually balancing the acceptance probabilities *P*_acc_(*k*). The *c* constant, along with the frequency of the scaling reset, regulates the speed of the balancing of the probabilities *P*_acc_(*k*). The probabilities *P*_acc_^*i*^(*k*) can be evaluated each few hundreds of attempted exchanges, and hence, with EAFs of the order of 20–30 ps^–1^, a good balancing may be obtained in a few ns or less with 1 < *c* < 2 and 0.001 × EAF < ω < 0.01 × EAF.

If *c* is selected to be too high, the algorithm quickly becomes unstable with corrections exceeding the scaling range. If this happens, then the iteration should be interrupted and restarted by resetting the HREM scaling ladder to the original protocol given by eq 3 and by decreasing *c*. The choice of parameter ω depends on the selected EAF which, in turn, is a function of the communication overhead between replicas in a parallel execution. In the GROMACS program^31^ patched with the PLUMED library,^48^ for example, the communication cost is high since configurations are exchanged among MPI processes. In ORAC,^23,45^ only the scaling factor is exchanged among replicas, with a minimal MPI overhead. For GROMACS, one should hence choose a smaller EAF (less frequent exchanges) and consequently a smaller ω by protracting the acceptance equalization scheme for a longer time until exchange equalization. Using the ORAC, the equalization algorithm is faster since EAF and omega can be selected large. The equalization period (i.e., the time needed for the variance σ^2^ of the exchange ratios to be below a given threshold) is a function of both *c* and ω. The larger the *c* and ω, the faster the on-the-fly equalization of the acceptance ratios.

Efficiency of the flow in an HREM simulation can be further assessed by measuring the fraction of the total simulation time that a replica spends in a given state. Ideally, each replica should spend the same fraction of time on each node/state given by τ(*k*) = 1/*n*. Deviation from the ideality can be measured by evaluating the quantity
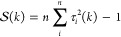
6where the sums are on all replica walkers.  corresponds to the variance of the variable τ_*i*_(*k*)/(1/*n*) expressing the ratio between the observed and ideal fractions of time the *i*-th replica walker has spent on the node *k*. Ideally, .

We conclude this section by recalling that, as stated in ref.,^44^ in complex systems long-term transition probabilities are usually different from observed acceptance rates, that are affected by short-time properties. As a consequence of this fact, balancing the acceptance ratio, while suppressing bottlenecks in the replica diffusion, may not necessarily lead to flux linearization or  minimization.

## 3 Methods

### 3.1 Compounds

**Figure 1:**
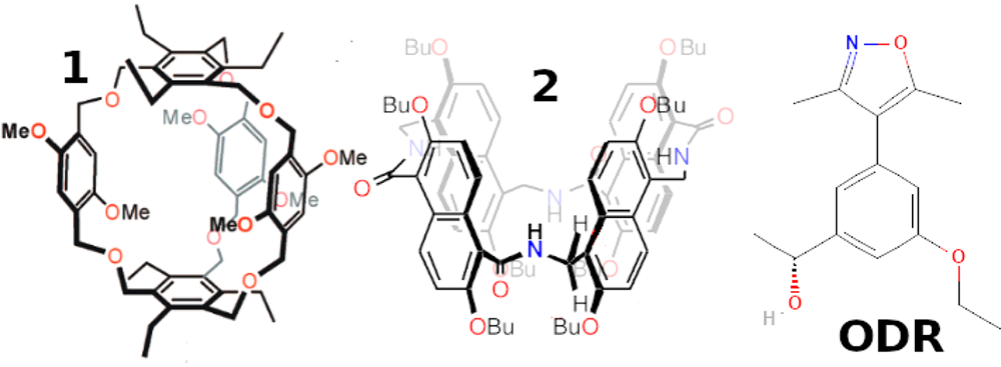
Macrocycles **1** and **2** and BRD4 ligand ODR.

In Figure 1, we show the structures of macrocycles **1** and **2** and of the ligand ODR of the first bromodomain of human BRD4. The structures of **1** and **2** were obtained from Cambridge Crystallographic Data Centre, CCDC 2051483^27^ and CCDC 1950443.^28^ Phenyl[5]-cage **1** is characterized by two rapidly exchanging structures due to the flipping of the three phenyl sidewalls.^27^ The two conformations can be distinguished in NMR experiments when **1** selectively binds to quarternary ammonium ions via an induced fit mechanism. Compound **2** (amide naphthotube) has five different conformations due to the flipping of the nonequivalent naphthyl moieties around the amide and aminomethyl junctions. These five conformations are reported in Figure 2e of ref.^28^ Experimentally, only two (out of five) conformations of the *apo* form of amide naphthotube in apolar solvents can be distinguished from NMR experiments, indicating that in **2** the exchange occurs in a time scale that exceeds that of NMR (ns to ms). The structures represented in Figure 1, taken from the Cambridge Structural Database, correspond to the most symmetric ones for compounds **1** and **2**.

Compound ODR is known to be a moderate binder of the BRD4(I) protein with an affinity of 4.8 μM.^49^ The initial structure of the complex was obtained from the 3svg PDB file.

We used the GAFF2 force field for the two macrocycles and the ligand ODR. Assignment and AM1-BCC^50^ charge calculations were done using the PrimaDORAC web interface.^51^ The BRD4 protein was modeled using the AMBER99SB-ildn force field.^52^

### 3.2 Simulation Details

Compounds **1** and **2** were simulated *in vacuo*. This was done to sample the full attainable conformational space without mediation of intercalated apolar solvent molecules (such as CHCl_3_ or C_2_H_2_Cl_2_) experimentally favoring the symmetric forms.^27^ For both **1** and **2**, simulations were hence conducted on a single macrocyclic molecule at constant temperature using a Nosé thermostat.^53^ The HREM simulations for compounds **1** and **2** were done using a protocol^23^ with scaling of all intramolecular interactions (except for stretching and bending) with *s*_min_ = 0.1 corresponding to a maximum “temperature” of 3000 K. We used eight or 12 states/replicas depending on the system, with the scaling protocol given by *s*_*k*_ = *s*_*min*_^(*k*–1)/(*n*–1)^ and an EAF of 20 ps^–1^. HREM produced 16 ns on the target state, for a total of 128 or 192 ns of simulations. In a second series of HREM simulations on compounds **1** and **2**, the scaling protocol was optimized in the first 1.6 ns by balancing the acceptance ratio on the fly using the iterative scheme of eq 4 (*c* = 2 and ω = 1/160 ps^–1^ with ten iteration cycles).

For the ODR-BRD4(I) complex, starting from the 3SVG PDB structure, we performed a pre-equilibration stage at the constant pressure of 1 atm and constant temperature of 300 K for 1 ns in a cubic box containing ≃7500 water molecules (modeled using the TIP3P potential).^54^ Constant pressure was enforced using a Parrinello–Rahman Lagrangian for isotropic stress tensor.^55^ Constant temperature was imposed using two Nosé thermostats coupled to the internal coordinates and center of mass coordinates of the molecules.^53,55^ Long range electrostatics were treated using the Particle Mesh Ewald technique.^56^ For the BRD4(I) complex, we used a solute-tempering HREM with a “hot zone” including the ODR ligand and the nearby residues (namely, MET43, TRP81, PHE83, GLN85, VAL87, LEU92, LEU94, TYR97, TYR139, ASN140, ASP145, ILE146, MET149). The HREM simulations were performed using 12 replicas for 24 ns on the target state (288 ns in total) with the standard scaling protocol *s*_*k*_ = *s*_*min*_^(*k*–1)/(*n*–1)^ with *s*_min_ = 0.1 and *n* = 12. In a second HREM simulation of the complex, the acceptance ratio was balanced on the fly for the first 1.6 ns. In eq 4, we used *c* = 2 and ω = 1/160 ps^–1^ with ten iteration cycles.

All simulations were performed using the program ORAC6.1^45^

## 4 Results

### 4.1 Compound **1**: Induced Fit

**Figure 2:**
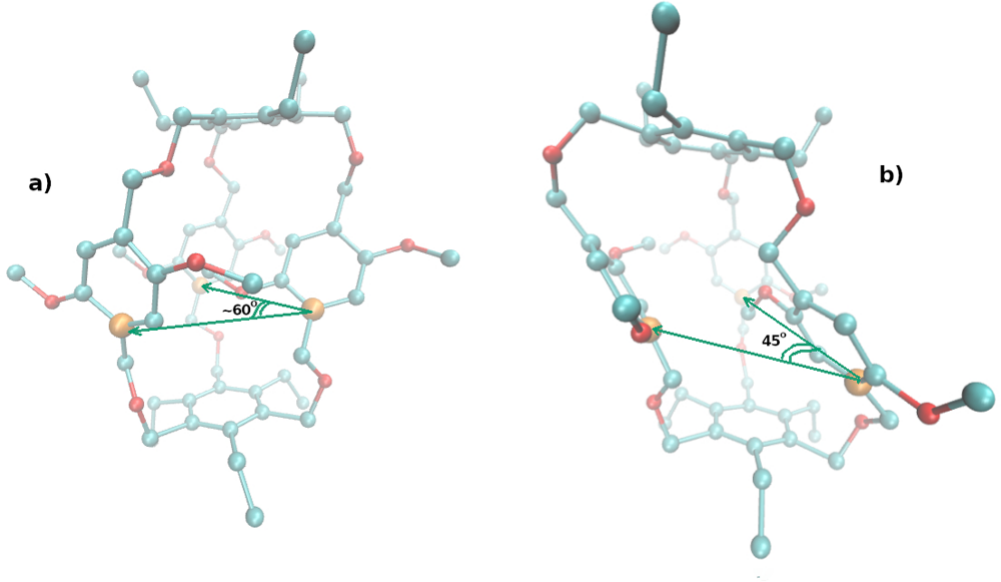
Main conformers in compound **1** in the gas-phase. a) Symmetric conformation; b) twisted conformation.

Compound **1** adapts its conformation to the binding guest via an induced fit mechanism.^27^ In Figure 2, we show the two main conformations of macrocycle **1**. The two cage structures can be distinguished by the value of any of the three angles α involving equivalent phenyl carbons (orange) on the three sides of the cage. In the most symmetric conformer **a** (on the left in Figure 2), the three angles are in the range 55–70 degrees, while in the twisted structure **b** (on the right in Figure 2), one of the angles is around 45 deg. One of the two rapidly exchanging conformations is in general selected when **1** binds specifically to quaternary ammonium cations.^27^

#### 4.1.1 HREM Simulations

We performed two HREM simulations, each lasting 16 ns, of compound **1***in vacuo* at 300 K. In both cases, due to the relatively low conformational barriers between the two structure (see Figure 4 further on), we used only eight replicas and a minimum scaling factor of 0.1. In one of the HREM simulations, the scaling protocol is given by eq 3. In the other, we used the acceptance ratio balancing algorithm of eq 4 starting from the default scaling protocol of eq 3. In Table 1, we report the acceptance probability (in percent) for the HREM simulations with and without *P*_acc_ balancing. In the standard HREM simulation, with the default scaling eq 3, *P*_acc_ balance is poor, with *P*_acc_ at the end of the ladder being more than 1 order of magnitude larger with respect to exchanges 1 ↔ 2 involving the target state. In the adaptive HREM simulation, excellent balancing was reached after ten iterations of eq 4 (taken at regular intervals in the first 1.6 ns simulation) at the expense of the mean acceptance ratio which dropped from 14.4% to 11.2%.

**Table 1:** Acceptance Probability (%) for the Unbalanced and Balanced HREM Simulations in Compound **1**

eq 4	1 ↔ 2	2 ↔ 3	3 ↔ 4	4 ↔ 5	5 ↔ 6	6 ↔ 7	7 ↔ 8
no	2.6	3.3	7.6	12.5	18.4	25.7	32.9
yes	8.0	10.5	12.4	12.7	11.8	11.2	11.4

As shown in ref.,^44^ the well balanced acceptance ratio, while avoiding bottlenecks in the replica diffusion, does not guarantee an ideal flux in the HREM simulation of conformationally complex systems. In Figure S1 of the Supporting Information (SI), we report the flux as a function of the nodes for the HREM simulations sampled during the 16 ns HREM simulations with (green) and without (dark green) the balancing of the scaling factors according to eq 4. As Figure S1 shows, the equalization of the acceptance ratio using eq 4 produced only a modest improvement on the flux. The mean absolute deviation with respect to the ideal flux is 0.06 and 0.09 for the balanced and unbalanced HREM simulations, evidencing the fact that well behaving short-time properties such as *P*_acc_ equalization do not necessarily imply an ideal or near-to-ideal flux across the replica progression.^44^ In Figure S2 of the SI, we show the results for variance  (see eq 6) obtained in the two HREM simulations. The variance  is similar to that of the standard HREM, with no significant gains when using the adaptive scheme based on *P*_acc_ equalization, hence confirming the moderate impact of *P*_acc_ on-the-fly balancing on replica flow.

Metrics for the two HREM simulations are finally collected in Table 2.

**Table 2:** HREM Metrics with and without *P*_acc_ Balancing in Compound **1**

eq 4	*N*_rep_	*s*_min_	⟨*P*_acc_⟩ (%)	*δP*_acc_	RTT (ns)	*Δf*_up_	
no	8	0.1	14.7	10.6	0.71	0.09	0.16
yes	8	0.1	11.2	1.4	0.62	0.06	0.22

As it can be seen, notwithstanding the fact that the standard deviation of *P*_acc_ is reduced by 1 order of magnitude, *P*_acc_ balancing has a marginal positive effect on the RTT and the deviation from the ideal flux and a negative effect on the mean acceptance ratio and . The benefit of ratio balancing via eq 4 for the replica flow is very likely negatively compensated by the significant decrease of the balanced mean acceptance ratio.

**Figure 3:**
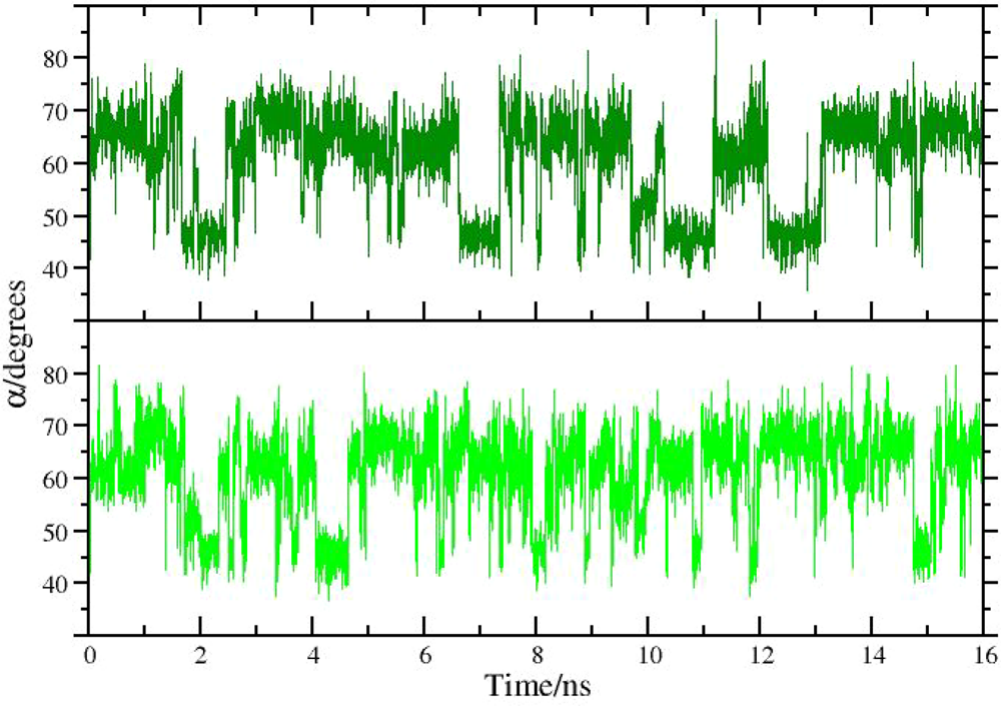
Time record of the α angle (see Figure 2) in an HREM simulation with fixed scaling of eq 3 (dark green) and with adaptive scaling according to eq 4 (green).

In Figure 3, we show the time record of angle α in the target state for two HREM simulations lasting 16 ns. Unlike in standard MD (see Figure 6 later), the swaps between the two conformations are frequent. In the HREM simulation with acceptance ratio balancing (bottom plot), swaps between the twisted and symmetric conformations appear to occur more frequently.

**Figure 4:**
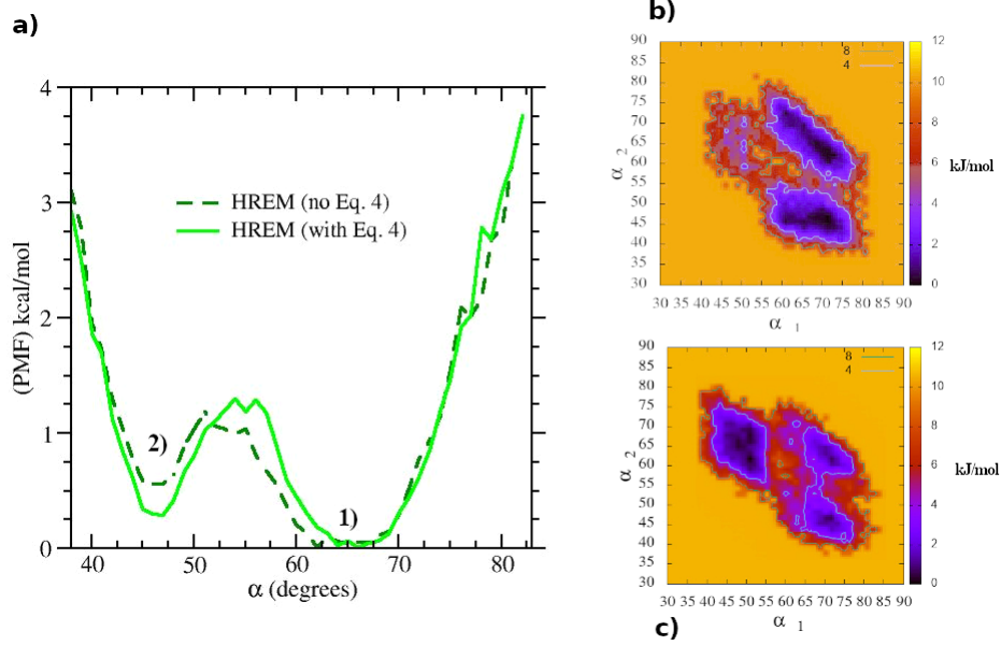
a) Potential of mean force along the α angle in compound **1**; green-dashed: standard HREM green: HREM with acceptance ratio balancing. The minima labeled 1) and 2) correspond to the structures of compound **1** shown in Figure 2. 2D PMF as a function of two angles (see text) computed by way of HREM without (b) and with (c) acceptance ratio balancing.

We finally examine the effect of eq 4 in the two HREM simulations with respect to the free energy difference between the two conformations of compound **1**, a quantity that is highly relevant for host–guest binding free energy calculations. To this end, in Figure 4a we report the calculated potential of mean force (PMF) along the α coordinate computed as *V*_PMF_(α) = −*RT* ln(*P*(α)/max(*P*(α)) where *P*(α) is the probability distribution of *one* of the three equivalent angles α defined by the three atoms highlighted in Figure 2 during the simulation of the gas-phase molecule. The green and dark green curves refer to the HREM simulation with and without *P*_acc_ balancing, respectively.

The two minima in the HREM simulations (referring to the symmetric and twisted conformations) differ by less than 0.2 kcal/mol, separated by a barrier of about 1 kcal/mol. The quality of the sampling in the two HREM simulations can be further assessed by evaluation of the two-dimensional PMF with respect to *any* pair of the angles of the triangle defined by the three atoms highlighted in Figure 2. Due to quasi-*C*_3*h*_ symmetry of structure **1** in Figure 2, we expect three minima for the cumulative 2D PMF with respect to any pair, with two equivalent minima with one angle of 45° corresponding to the twisted structure and wide minimum with angles in the range ≃55–70. The 2D PMF is reported in Figures 4b and 4c for the standard and acceptance ratio balanced HREM simulation, respectively. The latter PMF is closer to the expected landscape, showing that the quality of sampling has been somewhat enhanced by the use of eq 4.

#### 4.1.2 Standard MD Simulations

**Figure 5:**
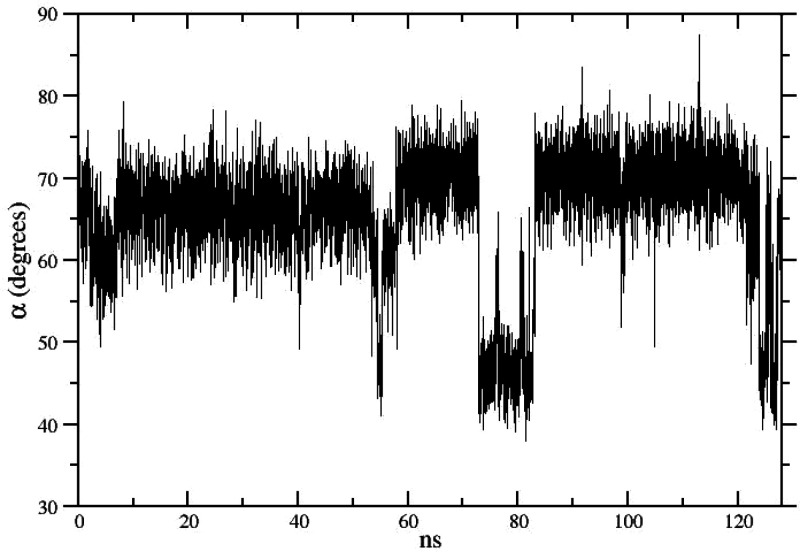
Time record of the α angle in compound **1** (see Figure 2) in a 128 ns standard MD simulation.

In Figure 5, we show the time records of one of the three α angles (see Figure 2) in a standard MD lasting for 128 ns, i.e., using the same amount of total CPU time of the HREM simulations previously described. No abrupt changes of the angle are observed during the first 50 ns of simulation implying that no flipping of the two involved phenyl moieties has taken place during this time.

Flipping in the first 50 ns occurs only on the third phenyl side probed by the other two α angles (data not shown). Note that phenyl flipping as probed by the angle occurs only three times during the whole 128 ns time span, in striking contrast (Figure 3) with the two HREM simulations where many angle swaps are observed in the target state in only 16 ns of sampling.

As authoritatively noted more that two decades ago, “individual [long MD] trajectories sample only a fraction of the conformational distribution generated by ten [much shorter] independent MD] trajectories”.^57^ This fact can be understood by considering that, while a single long trajectory may get stuck in one of the conformational attractors for a long time before it can jump to a different conformational state, many short trajectories started from infinitesimally different initial conditions have a better chance of concurrently sampling several attractors.^34,38^

**Figure 6:**
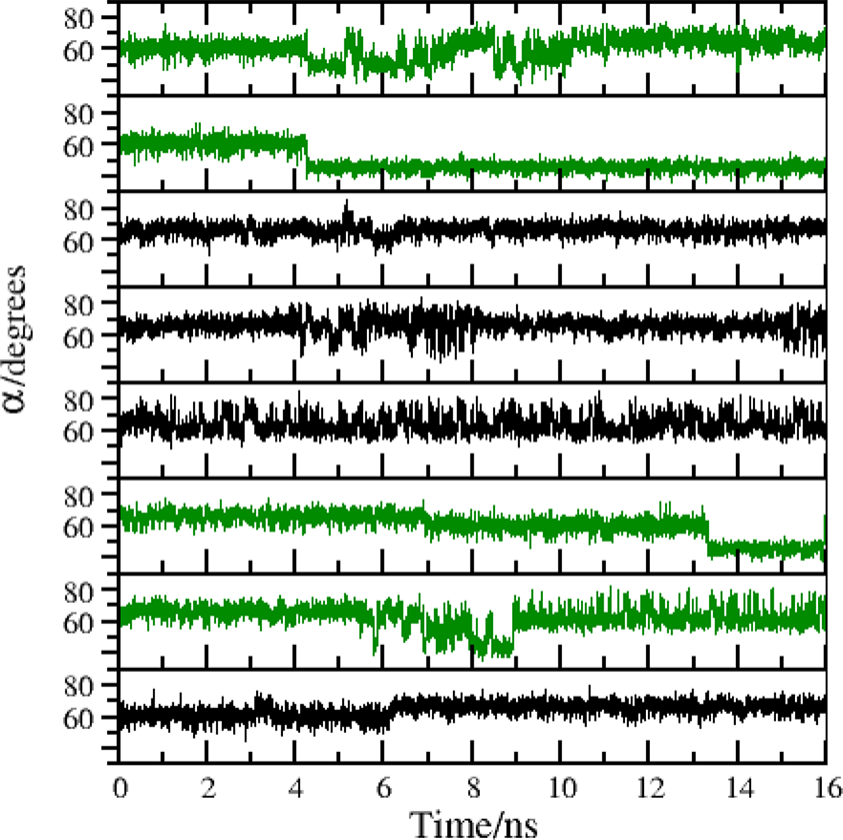
Eight replicates of 16 ns MD simulation of compound **1**.

In Figure 6, we show the time record of one of the three equivalent angles in the triangle of Figure 2 for eight *replicates* of standard MD simulations of compound **1** each lasting 16 ns, with a cumulative sampling time of 128 ns, i.e. the same amount of time of the standard MD simulation of Figure 5. The rare event of the switching of the two conformations, as probed by one of the α, has been observed six times in the cumulative 128 ns, compared to the three times observed only in the standard MD simulation of Figure 5.

**Figure 7:**
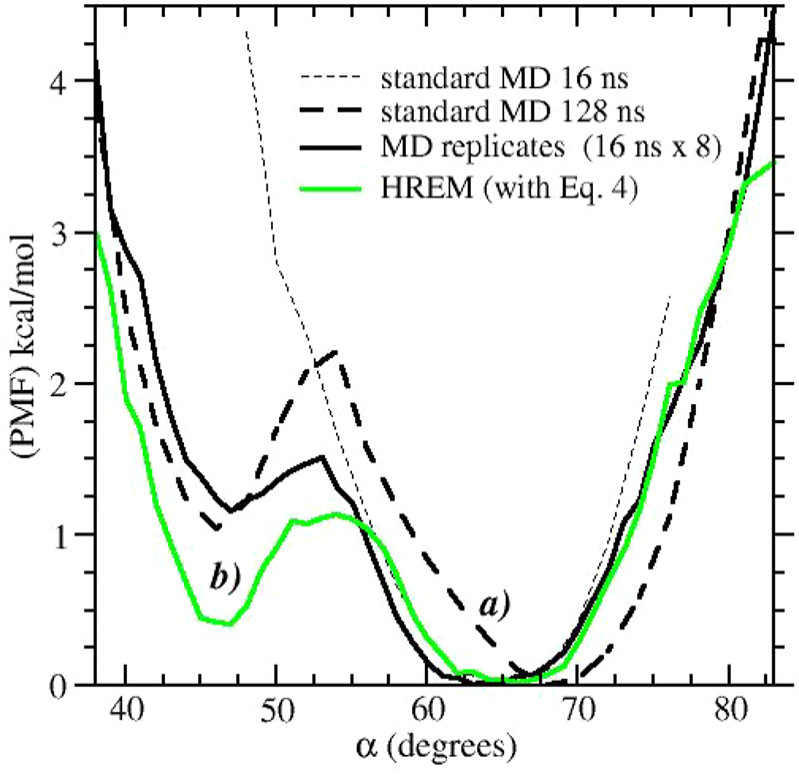
Potential of mean force along the α angle in compound **1** computed using standard molecular dynamics simulations (black color) and HREM using eq 4 (green color).

In Figure 7, we report the PMF with respect to the α angle obtained with standard MD simulations.

For comparison, we also included in the plot the PMF obtained using HREM with acceptance ratio balancing. None of the approaches based on standard MD are apparently able to produce fully reliable Boltzmann weights for the two conformations of compound **1** whose binding is characterized by the induced fit mechanism. We recall that in alchemical free energy simulations, a correct sampling of the *apo* state, or of states with small alchemical coupling, is an essential requirement for delivering reliable binding free energies.^58^ Phenyl flipping in compound **1**, as probed by α, never occurs in standard 16 ns MD simulations, while it is severely undersampled by extending the simulation up to 128 ns. The best results (i.e., those that are close to those obtained with the two HREM simulations) are obtained using 16 ns replicates of standard MD simulations confirming the moderate effectiveness of this straightforward enhanced sampling approach.^34,38^

### 4.2 Compound **2**: Conformational Selection

**Figure 8:**
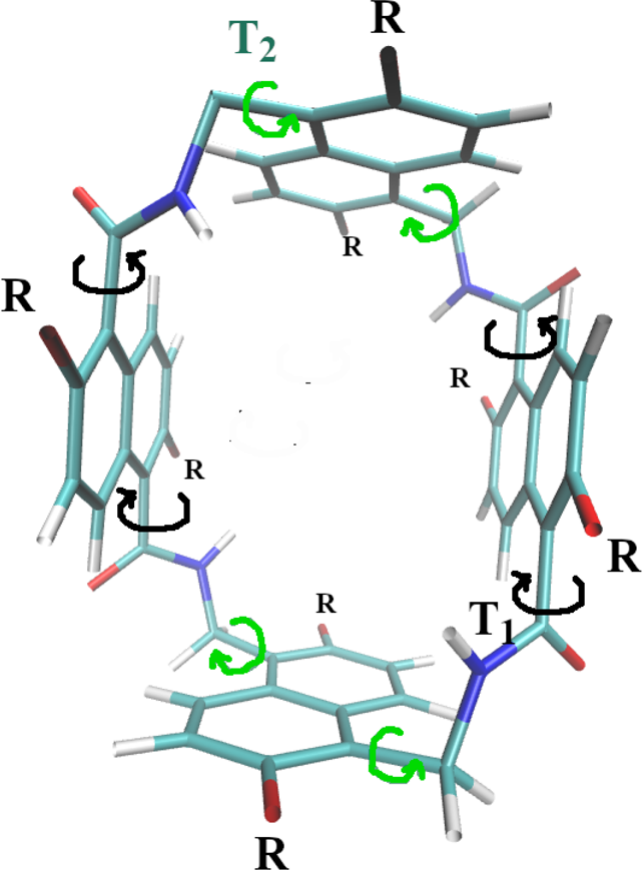
Compound **2** in configuration I with *C*_2_ symmetry.^28^ The torsions around sp_3_ bonds connecting methylene or amide moieties are shown in green and black colors, respectively.

Compound **2** is characterized by two nonequivalent couples of stacked naphthyl moieties whose flipping is regulated by torsions around two carbon–carbon sp_3_ bonds connected in one case to amide and in the other case to methylene moieties (see Figure 8). Because of the bulky naphthyl sides, torsional barriers for the switching between conformers are in the case of compound **2** much higher than they are in compound **1** with phenyl groups, hence giving rise to long-lived metastable conformational states determining the conformational selectivity of this host molecule.^28^

#### 4.2.1 HREM Simulations

Due to the presence of higher torsional barriers and because of a higher *N*, the HREM simulation of compound **2***in vacuo* required a minimum of 12 replicas with a minimum scaling factor of *s*_min_ = 0.1 to allow for at least one round trip on the average per replica during 16 ns of simulation (for a total invested HREM simulation of 192 ns). As for the case of compound **1**, we launched two HREM simulations with and without acceptance ratio balancing according to eq 4 with the starting standard scaling protocol given by eq 3. In Figure S3 of the SI, we show the flux (eq 2), the acceptance ratio, and the scaling protocols were obtained with the standard (eq 3) and adaptive (eq 4) HREM approaches. As observed for the case of compound **1** (see Sec. 4.1.1), the use of adaptive scheme eq 4 for acceptance ratio equalization leads to an overall decrease of the mean *P*_acc_ dropping to 18.3 from 28.0. Nonetheless, ratio equalization has a positive effect on the measured flux with a mean deviation from ideality (*Δf*_up_) decreasing to 0.09 from 0.07 of the standard HREM algorithm based on eq 3. Replica flow improvement with eq 4, while marginal for compound **1**, is significant in compound **2** where the torsional barriers for sidewall flipping are much higher than they are in host **1** (see Figure 10 further on). As shown in Table 3, where metrics for the HREM efficiency in the simulation of compound **2** are collected, the adaptive algorithm in eq 4 has a positive and significant effect on the mean round-trip time. The mean round-trip time is found to be 2.12 ns for the adaptive scheme, i.e., nearly 20% less that that obtained (2.68 ns) with the standard HREM scaling. Concerning variance  (eq 6), i.e. the mean deviation of the time spent on any given node/state by each walker with respect to the ideal fraction of 1/12, we observe an increase when using the adaptive scheme as already noticed for the case of compound **1** (see Table 1).

**Table 3:** HREM Metrics with and without *P*_acc_ Balancing in Compound **2**

eq 4	*N*_rep_	*s*_min_	⟨*P*_acc_⟩ (%)	*δP*_acc_	RTT (ns)	*Δf*_up_	
no	12	0.1	28.0	17.5	2.68	0.09	0.39
yes	12	0.1	18.3	3.2	2.12	0.07	0.50

**Figure 9:**
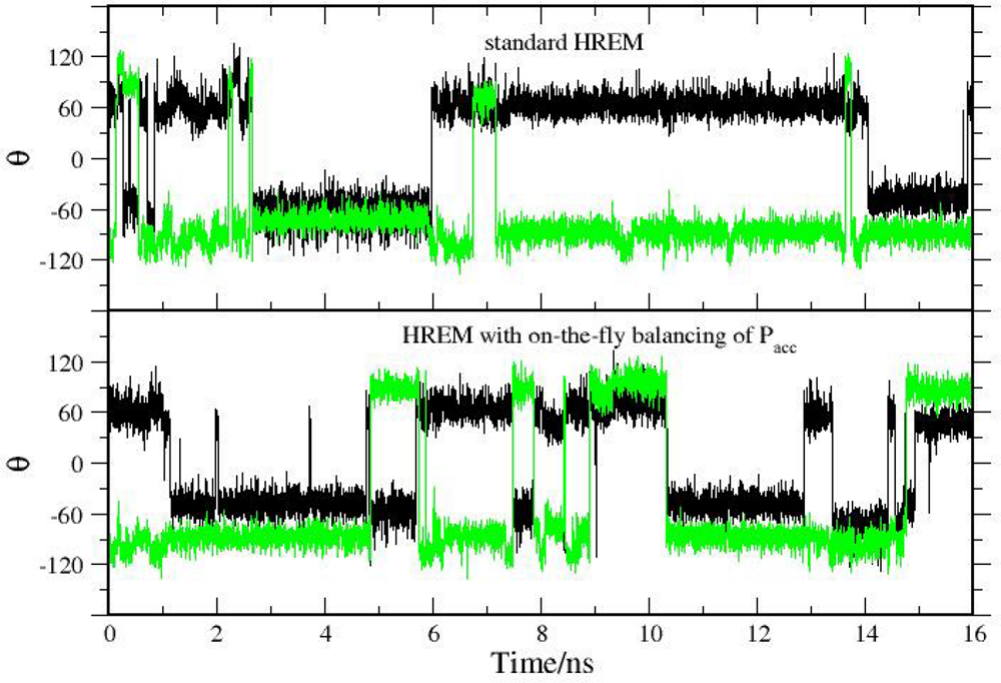
Time record of the *T*_1_ (black) and *T*_2_ (green) torsional angles (in degrees) in compound **2** (see Figure 8) for the HREM simulation with (bottom plot) and without (top plot) balancing of the acceptance ratio probability using eq 4.

In Figure 9, we show the time record of the dihedral angles *T*_1_ and *T*_2_ (see Figure 8) regulating the flipping of the two nonequivalent naphthyl moieties in compound **2** using HREM with and without the adaptive scheme of eq 4. As observed for compound **1**, flipping events are somewhat enhanced by using the adaptive scheme.

**Figure 10:**
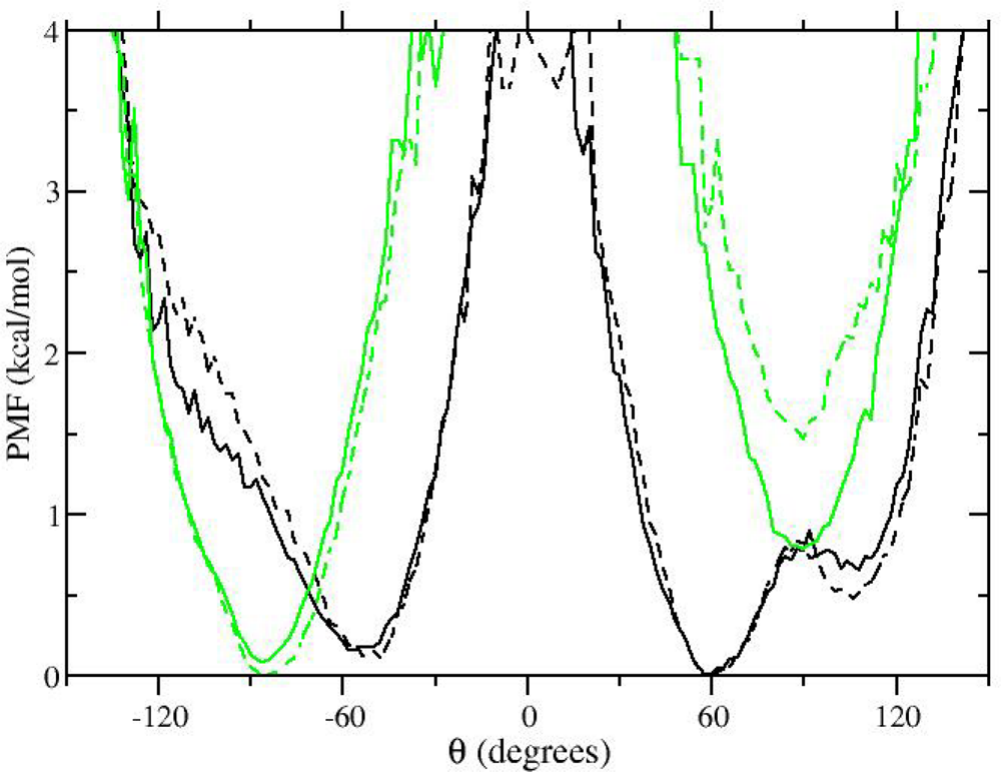
PMF of the *T*_1_ (black) and *T*_2_ (green) dihedral angles (see Figure 8) obtained with standard HREM (dashed lines) and with adaptive HREM using eq 4 for the *P*_acc_ balance.

In Figure 10, we finally show the PMF along the dihedral angles *T*_1_ and *T*_2_ obtained by using the two HREM protocols in the simulation of compound **2**. The two PMFs are quite similar, especially for the *T*_1_ torsion involving the naphthyl side walls connected by amide moieties (see Figure 8). For the *T*_2_ torsion, there are non-negligible discrepancies with the two minima differing by ≃1 kcal/mol in the case of the HREM with the adaptive scheme of eq 4 and ≃1.5 kcal/mol for the standard HREM simulation.

Given the better behavior of the flow and the shorter RTTs obtained in the case of HREM with the adaptive scheme, we can assume that the usage of eq 4 somewhat improves the sampling efficiency, hence providing a more accurate PMF with respect to the *T*_2_ collective coordinate. As expected, the torsional barriers for the flipping of the naphthyl groups exceed in both cases 4 kcal/mol, hence giving rise to long-lived metastable states in compound **2**. Quite surprisingly, in both HREM simulations, the higher barrier is detected for the *T*_2_ torsion involving the methylene moieties (see Figure 8).

#### 4.2.2 Standard MD Simulations

**Figure 11:**
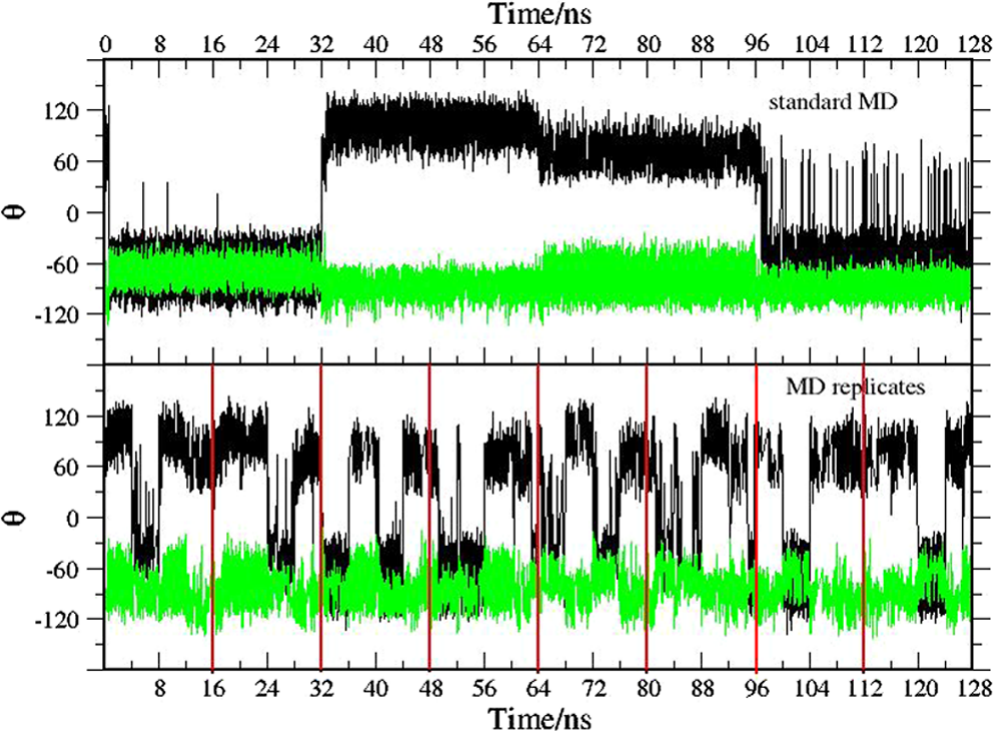
Time record of the *T*_1_ (black) and *T*_2_ (green) torsional angles (in degrees) in compound **2** (see Figure 8) for a standard MD simulation of 128 ns (top plot) and for 8 replicates of 16 ns standard MD (bottom plot). Red vertical lines mark the beginning and end of the MD replicates.

In Figure 11, we show the time record of the *T*_1_ and *T*_2_ dihedral angles during a 128 ns long standard MD simulation of compound **2**. We observed two naphthyl flipping as probed by *T*_1_ and no flipping for the methylene-connected naphthyl moieties during the entire simulation. Standard MD is apparently unable to reliably sample the conformational states of compound **2** even extending the simulation time beyond 100 ns. In the bottom plot of Figure 11, we show, sequentially, the time record of the *T*_1_ and *T*_2_ dihedral angles obtained in 8 MD simulation replicates, each lasting 16 ns only. Flipping of the *T*_1_ torsion is observed all the eight replicates, while again the *T*_2_ dihedral is stuck with the initial value of ≃−60° in each of the replicates. The rare event of naphthyl flipping occurs, as observed for the phenyl flipping in compound **1**, in a subpicosecond time scale.

**Figure 12:**
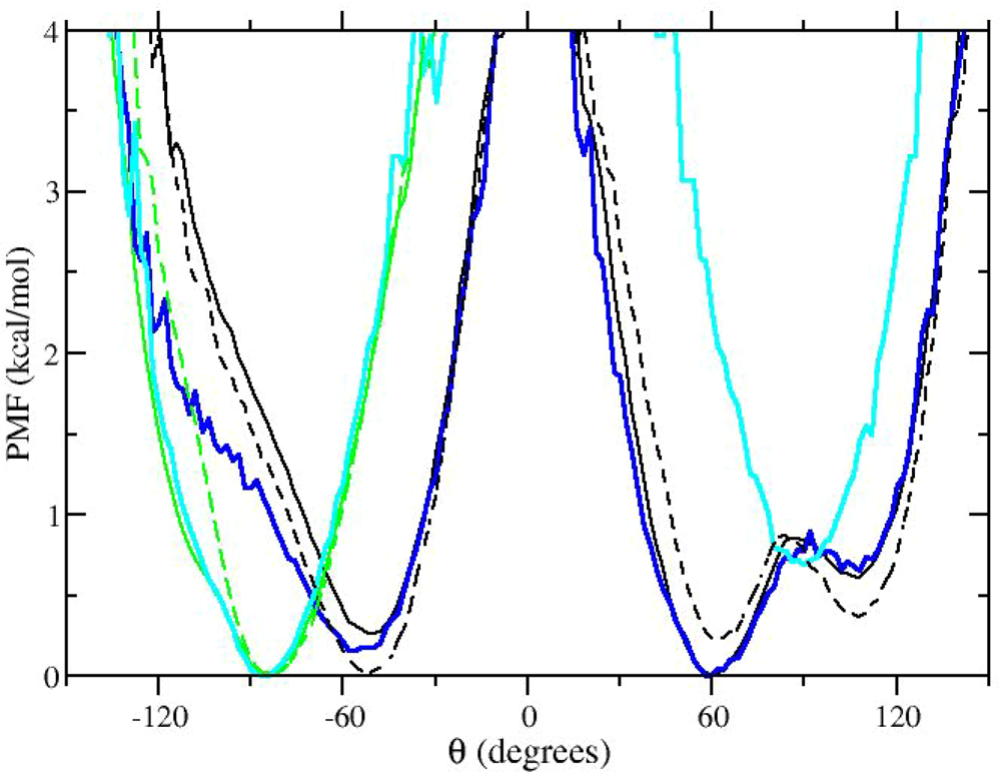
PMF of the *T*_1_ (black) and *T*_2_ (green) dihedral angles (see Figure 8) obtained with 128 ns long standard MD (dashed lines) and eight 16 ns long standard MD replicates (solid black and green lines). The PMF obtained by using the adaptive HREM simulation is shown in blue (*T*_1_) and cyan (*T*_2_).

In Figure 12, we finally show the PMF obtained for the torsional potential of the *T*_1_ (black color lines) and *T*_2_ (green color lines) dihedral angles using standard MD and replicates of much shorter standard MD simulations. In Figure 12, the PMF obtained with the adaptive HREM (in blue and cyan) is also superimposed for comparison. For the *T*_1_ torsion, characterized by a barrier of ≃5 kcal/mol, we notice that the statistics collected in the 8 replicate MD simulations (128 ns in total) produces a PMF that closely follows that obtained with the 16 ns of HREM simulation. For *T*_2_, standard MD simulations, whether using short replicates or a single long lasting run, are unable to sample the secondary minimum detected in HREM at θ = 60°, showing the limitations of the straightforward replicate approach when dealing with long-lived metastable states.

### 4.3 BRD4(I) in Complex with the ODR Ligand

**Figure 13:**
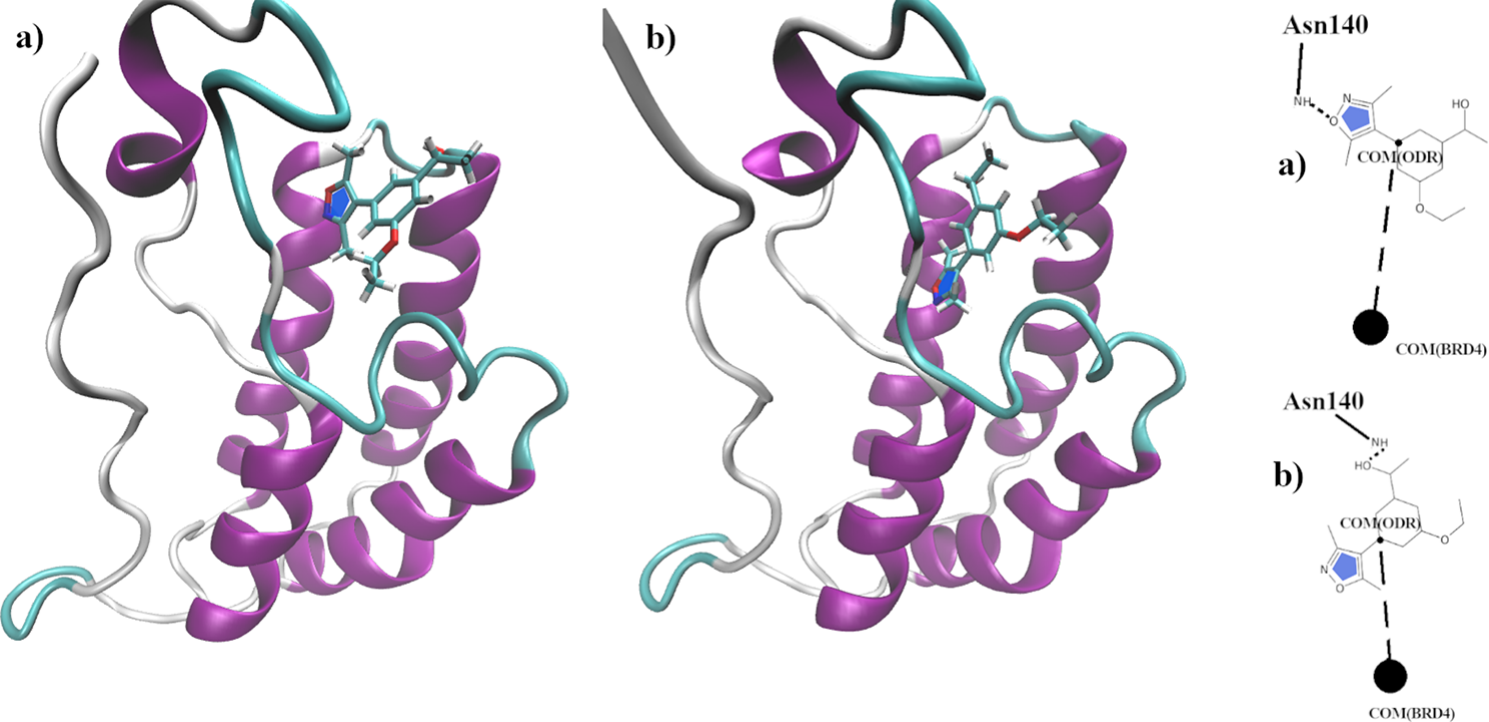
a) Crystallographic and primary pose of the ODR ligand in complex with BRD4(I); b) secondary pose of the ODR ligand sampled in the HREM and standard MD simulations.

We finally compare conformational sampling using standard HREM, HREM with acceptance ratio balancing (eq 4), and MD replicates for the simulation of BRD4(I) in complex with the ligand ODR (see Figure 1). Technical details of the simulation are provided in Sec. 3.2. In Figure 13, we show representative configurations of the two main poses of the ODR ligand in the BRD4(I) scaffold sampled during the MD runs. Labels a and b in Figure 13 refer to the crystallographic pose in the 3SVG PDB file and to the secondary poses observed in the simulations, respectively. The two poses are approximately related by a clockwise rotation of 60° with respect to an axis passing through the center of the ligand phenyl ring and perpendicular to it. As shown on the left of Figure 13, the primary pose a is characterized by a persistent H-bond between the oxygen atom of the isoxazole moiety of the ODR ligand and the hydrogen atom of the NH group in ASN140. Such an H-bond is no longer present in the secondary pose b, substituted by a much less stable H-bond between the NH group of ASN140 and the oxygen of the hydroxy group of the ligand.

**Figure 14:**
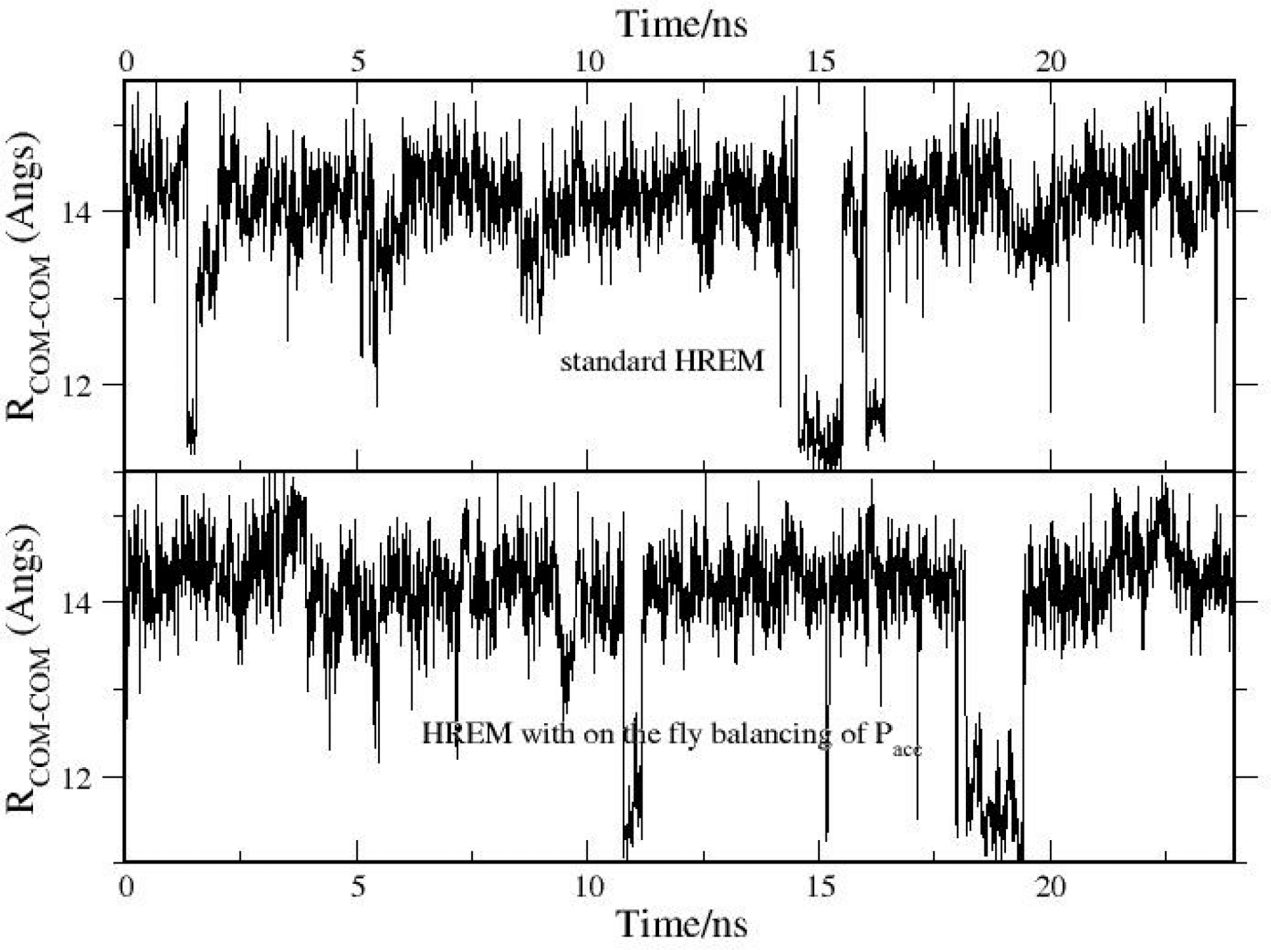
Time record of the distance between the center of mass of BRD4(1) and of ODR using standard HREM (top) and adaptive HREM using eq 4.

In Table 4, we have collected all relevant parameters for the HREM simulation of the ORD-BRD4(I) complex in an explicit solvent. The subsystem, involved in 12-fold scaling down to *s*_min_ = 0.1, includes the ligand and the hot-zone residues for a total of 183 atoms, comparable to the number of atoms in compound **2** (157), which was simulated using the same HREM protocol. As a trivial consequence of this fact, we observe the same pattern in the acceptance ratio using standard scaling eq 3 in the BRD4(I) complex and in compound **2** with *P*_acc_(*k*) steadily increasing with node index *k* and with nearly identical mean values (29.1% for BRD4(I)-ODR and 28% for compound **2**). As observed for the HREM simulations of compound **2**, also in the case of the BRD4(I)-ODR complex, the flux and RTT are slightly improved by implementing acceptance ratio balancing via eq 4. In the BRD4(I)-ODR complex, we observe only a moderate degradation of the mean acceptance ratio upon ten iteration cycles (lasting 1.6 ns in total) of implementing eq 4, at variance with what was observed in compounds **1** and **2** (see Tables 1 and 3). As in compounds **1** and **2**, variance  (eq 6) increases when using eq 4. In Figure 14,

**Table 4:** HREM Metrics with and without *P*_acc_ Balancing in the Complex of the Molecule of ODR-BRD4(I)

eq 4	*N*_rep_	*s*_min_	⟨*P*_acc_⟩ (%)	*δP*_acc_	RTT (ns)	*Δf*_up_	
no	12	0.1	29.1	13.3	1.60	0.13	0.36
yes	12	0.1	28.3	7.6	0.97	0.11	0.47

we report the time record of the distance between the centers of mass of the ligand and the receptor (*R*_COM–COM_) obtained in the HREM simulation with and without the use of the adaptive scheme of eq 4 for *P*_acc_ balancing. The plot shows a clear prevalence of the crystallographic pose (see Figure 13a) characterized by *R*_COM–COM_ ≃ 14–15 Å. The secondary pose at *R*_COM–COM_ ≃ 11–12 Å (see Figure 13b) is sampled five and seven times in the standard and the adaptive HREM simulations, respectively, with a probability around 7% in both cases.

**Figure 15:**
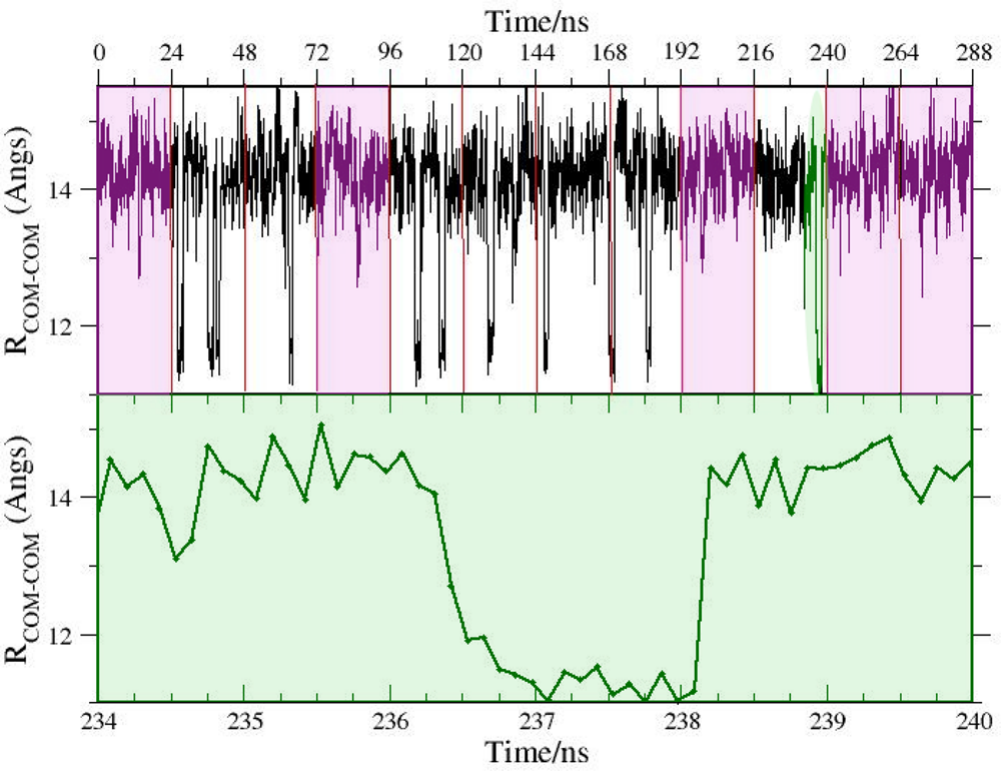
Time record of the distance between the center of mass of BRD4(1) and of the ODR using 12 replicates of the standard MD simulation, each lasting 24 ns.

In Figure 15, we report the time record of 12 juxtaposed replicates of standard MD simulations, each lasting 24 ns. The five magenta-highlighted replicates indicate independent 24 ns MD simulations where the secondary pose was never observed. The secondary pose was sampled 10 times in the total 288 ns time span of standard MD replicates, with a probability of 7% (as in HREM). In the bottom plot of Figure 15, we show a zoomed-in view of the 10th MD replicate where the pose switch was observed. Note that the effective duration of this rare event was less than 200 ps, despite the fact that the ligand must rotate by ≃60° around the axis perpendicular to the phenyl ring, breaking an H-bond and forming a new one (see Figure 13), resulting in an overall shift of the ligand COM of nearly 3 Å. Again, we are dealing also in this case with a rare event that occurs on a fast time-scale.

**Figure 16:**
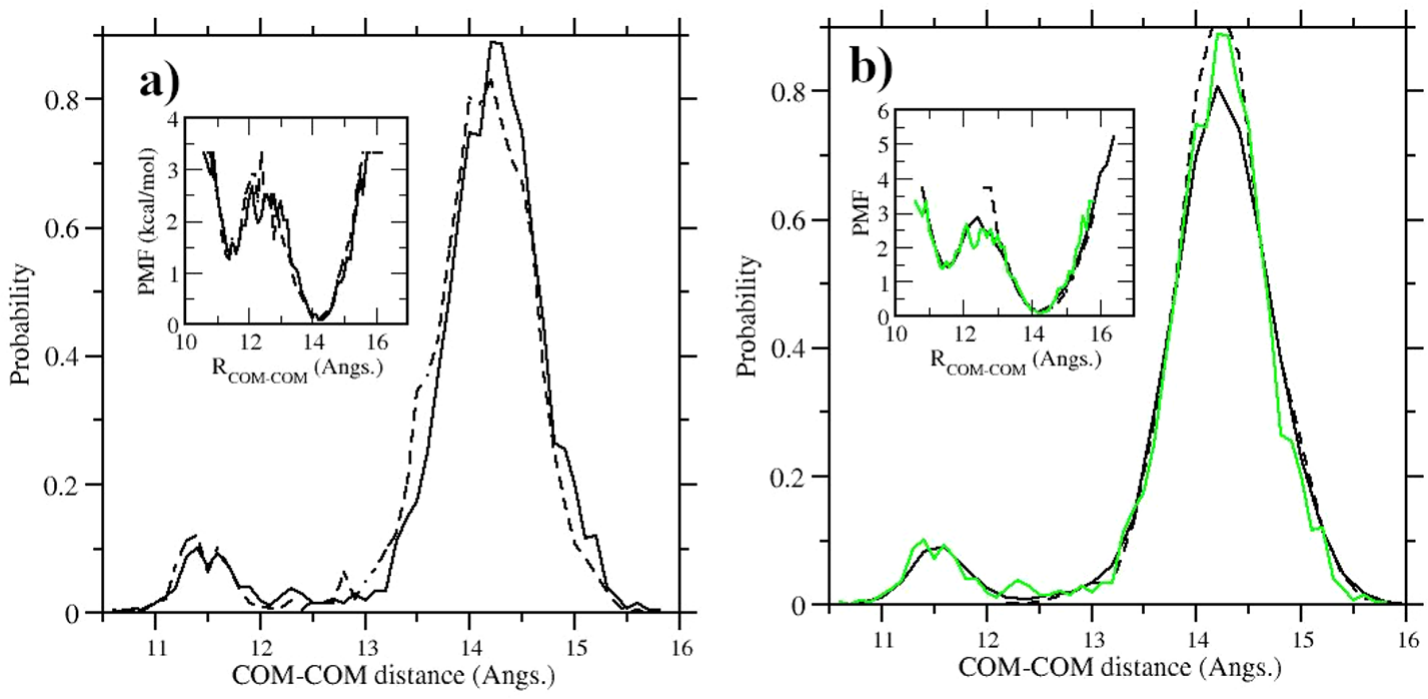
**a)** Ligand–receptor COM-COM distance distribution and associated PMF (in the inset) using standard HREM (dashed line) and HREM with adaptive scaling using eq 4 (solid line). HREM simulation lasted in both cases 24 ns; **b)** Ligand–receptor COM-COM distance distribution and associated PMF (in the inset) obtained with a 24 ns standard MD (dashed line) and with 12 replicates of 24 ns MD simulations (solid lines). In green, we show the distribution obtained with the adaptive HREM.

In Figure 16, we finally compare the probability distribution (and the associated PMF) of the poses of the ODR ligand in the BRD4(I) complex obtained in HREM and using MD. Inspection of Figure 16a shows that standard and adaptive HREM produces essentially the same probability profile and PMF. This probability and PMF can be recovered only using 12 replicates of MD (for a total of 288 ns), while a simple 24 ns vanilla simulation (first of the 12 replicates in Figure 15) completely missing the sampling of the secondary pose.

If on the one hand it is indeed encouraging that MD replicates are able to collectively sample the two ligand poses with the same probability obtained in HREM simulations investing the same amount of CPU time, then on the other hand, it is quite disheartening that we needed, in total, 288 ns of standard MD simulation for such results. In an FEP approach, there is no reason to believe that such amount of time is required *only* to sample effectively the initial state of the ligand–receptor complex. If we change the nature of the ligand receptor interactions, as occurs in the alchemical intermediate states of a standard FEP calculation, we very likely alter the probability ratio of the two poses, with an impact of the free energy change. For example, the oxygen of the isoxazole moiety of ODR is engaged in a strong hydrogen bond with a hydrogen of the NH_2_ group of ASN100 in the primary pose (see Figure 13). This H-bond is lost when the ligand switches to a secondary pose. Hence, when, e.g., the electric charges on the alchemical ligand are turned off, as commonly done in FEP calculations where electrostatic and Lennard-Jones interactions are sequentially switched off, the probability ratio of the two main poses in the BRD4(I)-ODR complex is likely to change significantly, with a direct effect on the free energy change. This fact is reported in Figure S4 of the SI where we computed, at a constant invested simulation time of 288 ns, the COM-COM distance distribution and corresponding PMF using HREM with and without acceptance ratio balancing and MD with replicates on the BRD4(I)-ODR system at a typical λ point in FEP protocols, i.e. that with the charge on the ODR molecule set to zero, so that the ligand in the bound state interacts with the environment only via the Lennard-Jones potential. For this particular alchemical state, the COM-COM distance distribution has two maxima with similar intensities corresponding to two binding poses with comparable strength, at variance with the disparate intensities observed in Figure 16. Note that as for the case of Figure 16 referring to the end state of the bound system, the COM-COM distribution using replicates of MD simulations is found to recover the HREM results.

The high cost required for a reliable sampling of any alchemical state, quantified in at least ≃300 ns for the BRD4(I)-ODR system whether using HREM or MD replicates, has a significant impact on the computational pipelines in drug-receptor binding free energy calculation using the well established FEP approach. Assuming 30 to 40 λ points in a standard FEP calculations of the bound state involving a relatively bulky ligand such as ODR implies that than no less of 10 μs of simulation time is needed for an FEP evaluation. On a high-end heterogeneous architectures such as the Leonardo cluster, recently deployed by the CINECA italian HPC consortium,^59^ a single FEP binding free energy calculation for a complex with 50k-60k atoms using GROMACS would engage for 24 h more than 60 nodes (each node has 4 Nvida A100 GPUS and 32 cores Intel Xeon)

In the MD-based alchemical approach for drug design, an alternative way of overcoming the formidable stumbling blocks in the sampling of complex conformational landscapes of hosts or ligand receptor systems is that of limiting the enhanced sampling simulations to the sampling of the end-states only and then, *in lieu* of the FEP λ stratification, connecting these end-states by a swarm of fast (a few ns at most) nonequilibrium (NE) alchemical simulations,^40,60,61^ recovering the free energy change from the resulting NE work distribution using the Jarzynski^62^ or the Crooks theorems.^63^ This approach, by forcing the occurrence of fast rare events exploiting the replica diffusion in the scaling ladder or the sampling efficiency of many short and independent MD replicates on parallel platforms, in some sense, restores the thermodynamics, hence avoiding the need to rely on an often unattainable ergodicity on the *each* intermediate alchemical state in a *single* extended simulation of a *single* molecular system characterized by a rugged free energy landscape.

## 5 Conclusion

We have performed extended MD simulations using Hamiltonian Replica Exchange and standard MD on two macrocyclic hosts, compound **1** (see Figure 2) and compound **2** (see Figure 8) that selectively bind small guest molecules via an induced fit and a conformational selection mechanism, respectively. HREM and MD simulations have also been performed on the complex of the ODR-BRD4(I) in TIP3 water, with the ligand occupying two competing poses (see Figure 13). In all cases, we used standard HREM based on the scaling protocol defined in eq 3 and an adaptive HREM with acceptance ratio balancing implemented in eq 4. Adaptive HREM seems to have a moderate positive impact on the average round-trip time and on the replica flux, resulting in a marginal effect on the sampling efficiency. “One off” MD simulations, even with a total simulation time exceeding by 1 order of magnitude that of the HREM simulations, are unable to reliably sample the conformational space in the two macrocyclic hosts and in BDR4(I)-ODR complex. By distributing the total simulation time in a series of shorter independent standard MD replicates, the sampling efficiency is restored in the case compound **1** and in the BRD4(I)-ODR complex where conformational substates or secondary poses are separated by free energy barriers not exceeding 1–2 kcal/mol. For compound **2**, characterized by long-lived metastable structures separated by barriers exceeding 4 kcal/mol, HREM appears to be the only viable alternative for reliable canonical sampling. The present results show that in complex systems with a manifold of possible conformations, a fully converged and reproducible FEP-based approach would require costly HREM simulations on *each* of the intermediate alchemical states. This severe sampling issue can be in part circumvented by limiting the enhanced sampling to the end-states alone (the complex with the fully coupled ligand and the receptor in the *apo* structure) and recovering the binding free energy via nonequilibrium fast alchemical transformations connecting these end states.

## Data Availability

PDB trajectory files from MD and HREM simulation (target state only), ORAC input files and force field parameter files are available at the general-purpose open-access repository Zenodo (https://zenodo.org/records/10003600). A beta version of the ORAC program with implementation of eq 4 in the rem module is also provided as a compressed tar archive in the same Zenodo repository.

## Supporting Information Available

Flux, variance  (see eq 6), and acceptance ratio for the HREM simulations of compounds **1** and **2**. COM-COM distance distribution and PMF at a λ intermediate point in the BRD4(I)-ODR complex using HREM and MD simulations (PDF)
