# Supplementary material for: Dealing with Induced Fit, Conformational Selection, and Secondary Poses in Molecular Dynamics Simulations for Reliable Free Energy Predictions
Source: J Chem Theory Comput. 2023 Dec 1;19(23):8942–54. doi: 10.1021/acs.jctc.3c00867 (PMC10720345; doi:10.1021/acs.jctc.3c00867)
Supplement: Supplementary file 1 — ct3c00867_si_001.pdf [file ct3c00867_si_001.pdf]

Supporting Information of "Dealing with induced fit, conformational selection and secondary poses in molecular dynamics simulations for reliable free energy predictions"

Piero Procacci*

Dipartimento di Chimica "Ugo Schiff", Università degli Studi di Firenze, Via della Lastruccia 3, 50019 Sesto Fiorentino, Italy

E-mail: piero.procacci@unifi.it

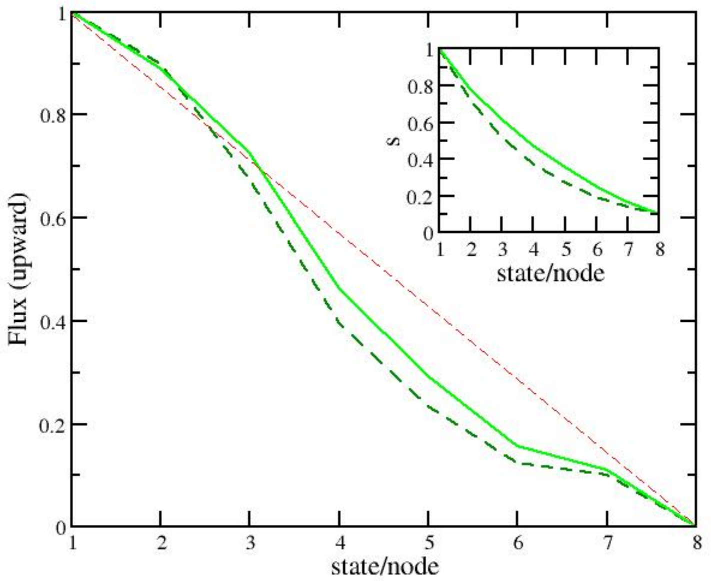

Figure S1: Upward flux (Eq. 2 of the main paper) in the HREM simulations of compound **1** estimated using the MFPT (see Section 2 of the main paper). Dashed dark green: standard HREM with fixed scaling (Eq. 3 of the main paper). Solid green: HREM with iterative acceptance ratio balancing (Eq. 4 of the main paper). In the inset we report the scaling factors as a function of the nodes with an without the adaptive scheme.

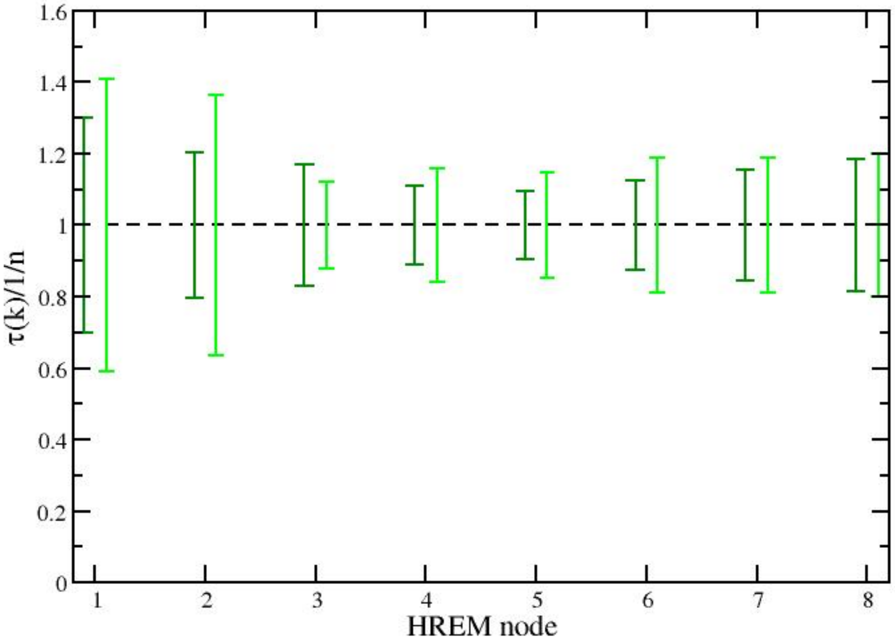

Figure S2: Variance as a function of the node index of the ratio $\tau(k)/(1/n)$ (see Eq. 6 of the main paper) in unbalanced (dark green) and balanced (green) HREM simulations of compound **1**

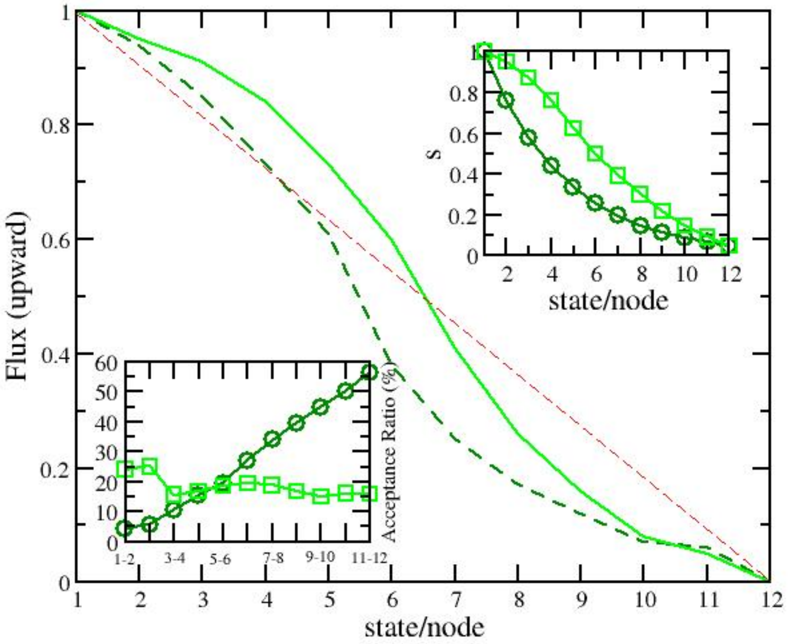

Figure S3: Upward flux (Eq. 2 of the main paper) in the HREM simulations of compound **2** estimated using the MFPT (see Sec. 2 of the main paper). Dark green: standard HREM with fixed scaling (Eq. 3 of he main paper). Solid green: HREM with acceptance ratio balancing (Eq. 4 of the main paper). In the top-right inset we report the scaling factors as a function of the nodes with an without the adaptive scheme of Eq. 4 of the main paper . In the bottom left inset, we report the acceptance ratios with and without the adaptive scheme of Eq. 4 of the main paper

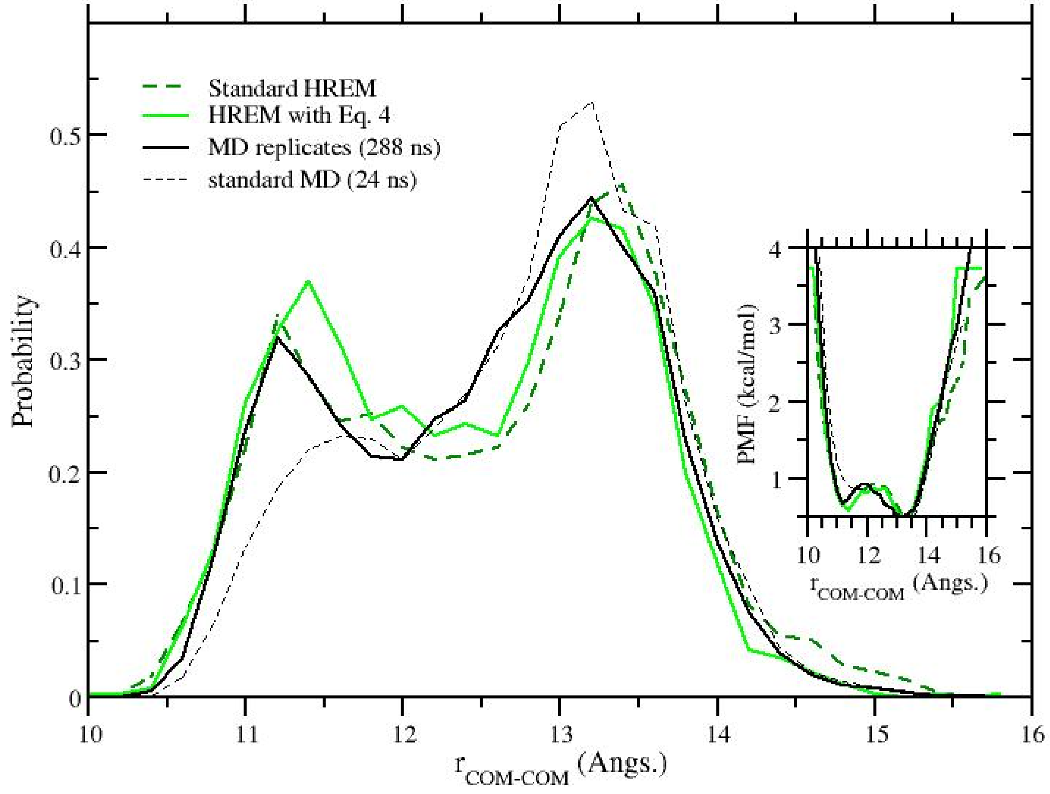

Figure S4: Ligand-receptor COM-COM distance distribution and associated PMF (in the inset) at the λ point with ligand-environment electrostatic interactions turned off computed using standard HREM (dark green dahsed line), HREM acceptance ratio equalization using Eq. 4 of the main paper (solid green line), and with 12 replicates of MD simulations each lasting 24 ns (solid black line).
